# Inflammatory plasma proteins predict short-term mortality in patients with an acute myocardial infarction

**DOI:** 10.1186/s12967-022-03644-9

**Published:** 2022-10-08

**Authors:** T. Schmitz, E. Harmel, M. Heier, A. Peters, J. Linseisen, C. Meisinger

**Affiliations:** 1 Epidemiology, Medical Faculty, University of Augsburg, University Hospital Augsburg, Stenglinstraße 2, 86156 Augsburg, Germany; 2 Department of Cardiology, Respiratory Medicine and Intensive Care, University Hospital Augsburg, Augsburg, Germany; 3 University Hospital of Augsburg, KORA Study Centre, Augsburg, Germany; 4 Helmholtz Zentrum München, Institute for Epidemiology, Ingolstädter Landstr. 1, 85764 Neuherberg, Germany; 5 Chair of Epidemiology, Institute for Medical Information Processing, Biometry and Epidemiology, Medical Faculty, Ludwig-Maximilians-Universität München, Munich, Germany; 6 German Center for Diabetes Research (DZD), Neuherberg, Germany; 7 German Research Center for Cardiovascular Research (DZHK), Partner Site Munich Heart Alliance, Munich, Germany

**Keywords:** Inflammatory marker, Myocardial infarction, 28-day mortality

## Abstract

**Background:**

The aim of this study was to investigate the association between inflammatory markers and 28-day mortality in patients with ST-elevation myocardial infarction (STEMI).

**Methods:**

In 398 STEMI patients recorded between 2009 and 2013 by the population-based Myocardial Infarction Registry Augsburg, 92 protein biomarkers were measured in admission arterial blood samples using the OLINK inflammatory panel. In multivariable-adjusted logistic regression models, the association between each marker and 28-day mortality was investigated. The values of the biomarkers most significantly associated with mortality were standardized and summarized to obtain a prediction score for 28-day mortality. The predictive ability of this biomarker score was compared to the established GRACE score using ROC analysis. Finally, a combined total score was generated by adding the standardized biomarker score to the standardized GRACE score.

**Results:**

The markers IL-6, IL-8, IL-10, FGF-21, FGF-23, ST1A1, MCP-1, 4E-BP1, and CST5 were most significantly associated with 28-day mortality, each with FDR-adjusted (false discovery rate adjusted) p-values of < 0.01 in the multivariable logistic regression model. In a ROC analysis, the biomarker score and the GRACE score showed comparable predictive ability for 28-day mortality (biomarker score AUC: 0.7859 [CI: 0.6735–0.89], GRACE score AUC: 0.7961 [CI: 0.6965–0.8802]). By combining the biomarker score and the Grace score, the predictive ability improved with an AUC of 0.8305 [CI: 0.7269–0.9187]. A continuous Net Reclassification Improvement (cNRI) of 0.566 (CI: 0.192–0.94, p-value: 0.003) and an Integrated Discrimination Improvement (IDI) of 0.083 ((CI: 0.016–0.149, p-value: 0.015) confirmed the superiority of the combined score over the GARCE score.

**Conclusions:**

Inflammatory biomarkers may play a significant role in the pathophysiology of acute myocardial infarction (AMI) and AMI-related mortality and might be a promising starting point for personalized medicine, which aims to provide each patient with tailored therapy.

**Supplementary Information:**

The online version contains supplementary material available at 10.1186/s12967-022-03644-9.

## Introduction

Prior studies investigated the prognostic performance of several biomarkers in acute myocardial infarction (AMI) patients [1–6]. Nevertheless, these studies mainly concentrated on nowadays well-known biomarkers like cardiac troponin I, N-terminal pro B-type natriuretic peptide (NT-proBNP), C-reactive protein, lipoprotein (a) and many more. These markers play important roles in the pathophysiology and diagnosis of AMI. Due to the complexity of underlying pathophysiology in coronary artery disease (CAD), neither occurrence nor progression of CAD is understood completely. It is likely that additional pathophysiological processes are involved in the development of CAD or the occurrence of an AMI and its outcome respectively. In this context, science focusses on the detection and measurement of biomarkers that can differentiate between physiological and pathological processes in the body [7–9]. So the aim of this study was to identify previously unknown protein biomarkers that might be associated with short-term mortality after AMI and evaluate these with already known predictors.

## Methods

### Study population

This study was based on data from the population-based Augsburg Myocardial Infarction Registry which was established in 1984 as a part of the MONICA-project (Monitoring Trends and Determinants in Cardiovascular disease) and since then operated as KORA (Kooperative Gesundheitsförderung in der Region Augsburg) Myocardial Infarction Registry [10]. The study area consists of the city of Augsburg, Germany, and the two adjacent counties comprising a total of approximately 680,000 inhabitants. All patients aged between 25 and 84 years being admitted to one out of eight hospitals in the study area were consecutively registered. More detailed information on case identification, diagnostic classification of events and quality control of the data can be found in previous publications [10, 11]. For the present study blood samples were taken from patients with ST-elevation myocardial infarction admitted to the university hospital of Augsburg between May, 2009 and July, 2013 (blood from patients was collected solely in this hospital). Out of all patients from which blood samples were taken, biomarkers of 398 consecutive patients were measured (mean age: 63.5 years (SD: 11.9), male: 73.1%). For 3 patients there were missing values for the biomarkers, thus 395 cases were included in the analysis. All study participants gave written informed consent. Methods of data collection were approved by the ethics committee of the Bavarian Medical Association (Bayerische Landesärztekammer) and the study was performed in accordance with the Declaration of Helsinki.

### Data collection

Trained study nurses interviewed the participants during hospital stay using a standardized questionnaire. To confirm the information provided by the patients and to collect additional information, the patients’ medical chart was reviewed. Demographic data, data on cardiovascular risk factors, medical history, comorbidities (including diabetes), medication before and during hospital stay, as well as at discharge were collected from each patient. Furthermore, laboratory parameters including glucose measurement, ECG and in-hospital course were determined.

Between 2009 and 2014 plasma samples were obtained within the scope of cardiac catheterization, which was in general performed immediately after hospital admission. Right at the beginning of the catheterization, EDTA blood samples were taken (arterial blood). This was immediately followed by the processing of the blood samples in the catheter laboratory (centrifugation, aliquoting and freezing at − 80 °C).

### Clinical chemistry measurement

Protein measurements of the 92 biomarkers were performed on plasma samples using the Proseek® Multiplex Inflammation panel, developed by Olink Proteomics (Uppsala, Sweden) and based on the Proximity Extension Assay (PEA). In the supplementary material, we give a brief description of the methods used for protein level quantification. Further information on the process of measurement can be found in a previous publication [12] and directly at the website of Olink Proteomics [13]. The selection of the 92 biomarkers of the inflammation panel was predetermined by Olink Proteomics and can´t be chosen individually.

All other blood parameters were measured in clinical laboratory at the university hospital of Augsburg during hospital stay of the patients as part of the regular diagnosis and routine treatment.

### Outcome

The endpoint used in this study was 28-day all-cause mortality. Information on mortality was obtained from the patients’ medical charts as well ascertained by regularly updates on the vital status of all registered persons of the MI registry with data from the population registries. Death certificates were obtained from local health departments.

### Statistical analysis

For the comparison of categorical variables, Chi-square tests were performed and the results were presented as absolute frequencies with percentages. For normally-distributed continuous variables, Student’s t-tests were used. For continuous variables that were not normally-distributed we used nonparametric tests. The results are presented as mean and standard deviation (SD) or median and inter-quartiles range (IQR).

### Logistic regression models

First, we standardized the values for each biomarker (we centered and normalized the variable so that the transformed variable had an expectancy value of 0 and a statistical variance of 1 for every biomarker). The standardization provides comparability between the 92 biomarkers. To examine the associations between the biomarkers and 28-day mortality, we calculated 92 logistic regression models, one for every biomarker. The first models were adjusted for sex and age. To control the effect of multiple testing, we FDR-adjusted the obtained p-values. In a subsequent step, we calculated the same logistic regression models and adjusted for sex, age, renal function according to GFR, diabetes mellitus, hypertension, hyperlipidemia and PCI (p-values also FDR-adjusted).

Based on these models, we identified biomarkers that were strongly associated with 28-mortality (FDR-adjusted p-value < 0.01). We added the standardized values of the selected biomarkers to obtain a summed value, which then was used as a prediction score. To assess the predictive ability of the score, we compared it to the established GRACE score [14] serving as a reference. As we did not have all information in the exact defined manner, we tried to replicate the score as best as possible (for details see supplementary material). There were two main deviations from the original score: no information on Killip class was available, but we had information on left ventricular ejection fraction (LVEF), which we used instead. Furthermore, as only ST-elevation myocardial infarction cases were included in this study, every case was assigned to the ST deviation group. There were cases with missing values for the variables LVEF, cardiac arrest, elevated heart enzymes and heart rate at admission. As just ignoring these cases could potentially cause a selection bias, we conducted multiple imputation by chained equations. The number of iterations was 5 and the number of created imputed data sets was 5 as well. The imputation process was performed with MICE-package (R statistic software). The subsequent analyses were calculated with the pooled results from the imputed data sets.

Subsequently, we calculated a combined score (GRACE score and biomarker score). Therefore, we standardized both scores (GRACE and the new developed biomarker score) and added both together. For the calculation of the biomarker score, 3 out of 398 patients had missing values. The missing values required to calculate the GRACE score were imputed (36 patients had at least one missing value for a required variable). Consequently, 395 cases were included for the ROC curve analysis of the GRACE score and combined total score.

### ROC analysis

The predictive ability of the three scores—the new biomarker score, the original GRACE score and combined total score—was compared. We performed ROC analyses and calculated AUC for each score and compared the results using bootstrapping. Finally, we calculated continuous net reclassification improvement (cNRI) and integrated discrimination increment (IDI) to further compare the predictive abilities.

## Results

Table 1 displays the baseline characteristics for the total sample and stratified for 28-day survival.

**Table 1:** Baseline characteristics for the total sample and stratified 28-day survival

	Total sample (n = 398)	28 days survived (= 370)	Died within 28 days (n = 28)	p-value	Missing values
Age (mean, SD)	63.5 (11.9)	63.1 (11.9)	69.5 (10.7)	0.0056	0
Male	291 (73.1)	272 (73.5)	19 (67.9)	0.6673	0
Comorbidities					
Hypertension	303 (76.1)	279 (75.4)	24 (85.7)	0.3154	0
Diabetes mellitus	107 (26.9)	97 (26.2)	10 (35.7)	0.3832	0
Hyperlipidemia	219 (55)	208 (56.2)	11 (39.3)	0.1237	0
BMI (kg/m^2^)	26.8 (24.3–29.9)	26.9 (24.4–29.8)	26.8 (24.1–30.3)	0.995	24
BMI > 30 kg/m^2^	88 (23.5)	84 (23.2)	4 (33.3)	0.6398	24
Smoking status				0.324	25
Current smoker	157 (42.1)	149 (41.4)	8 (61.5)		
Ex-smoker	106 (28.4)	103 (28.6)	3 (23.1)		
Never smoker	110 (29.5)	108 (30)	2 (15.4)		
Clinical characteristics					
Prehospital time in minutes (median, IQR)	115 (198)	120 (212.5)	77.5 (57)	0.002716	29
Prehospital cardiac arrest	24 (6.2)	17 (4.7)	7 (26.9)	< 0.001	11
Systolic blood pressure at admission (median, IQR)	140 (125–160)	144.0 (129.25–160)	127.5 (93.5–140)	< 0.001	0
Diastolic blood pressure at admission (median, IQR)	80 (68.5–95.5)	80 (70–96)	67 (60–72.5)	< 0.001	3
Heart rate at admission (median, IQR)	76 (66–89)	76 (66–88.25)	80 (64.5–90)	0.4599	3
Left ventricular EF				< 0.001	16
> 50%	180 (47.1)	172 (47.8)	8 (36.4)		
41–50%	85 (22.3)	83 (23.1)	2 (9.1)		
31–40%	88 (23)	84 (23.3)	4 (18.2)		
≤ 30%	29 (7.6)	21 (5.8)	8 (36.4)		
Kidney function				< 0.001	0
eGFR ≥ 60 ml/min/1.73 m2	275 (69.1)	266 (71.9)	9 (32.1)	-	-
eGFR 30—59 ml/min/1.73 m2	109 (27.4)	91 (24.6)	18 (64.3)	-	-
eGFR < 30 ml/min/1.73 m2	14 (3.5)	13 (3.5)	1 (3.6)	-	-
Treatment					
PCI	363 (91.2)	340 (91.9)	23 (82.1)	0.1585	0
Bypass therapy	36 (9.7)	36 (9.7)	5 (17.9)	0.2975	0
Lysis therapy	3 (0.8)	2 (0.5)	1 (3.6)	0.5126	0
Laboratory values					
Troponin I at admission* (median, IQR)	0.59 (0.09–5.91)	0.56 (0.09–4.975)	3.41 (0.2875–12.275)	< 0.001	7
peak CRP (median, IQR)	0.38 (0.29–1.0325)	0.370 (0.29–0.975)	0.575 (0.29–1.5425)	0.474	2

^*^Cut off value of the labaratory was 0.14

**Fig. 1:**
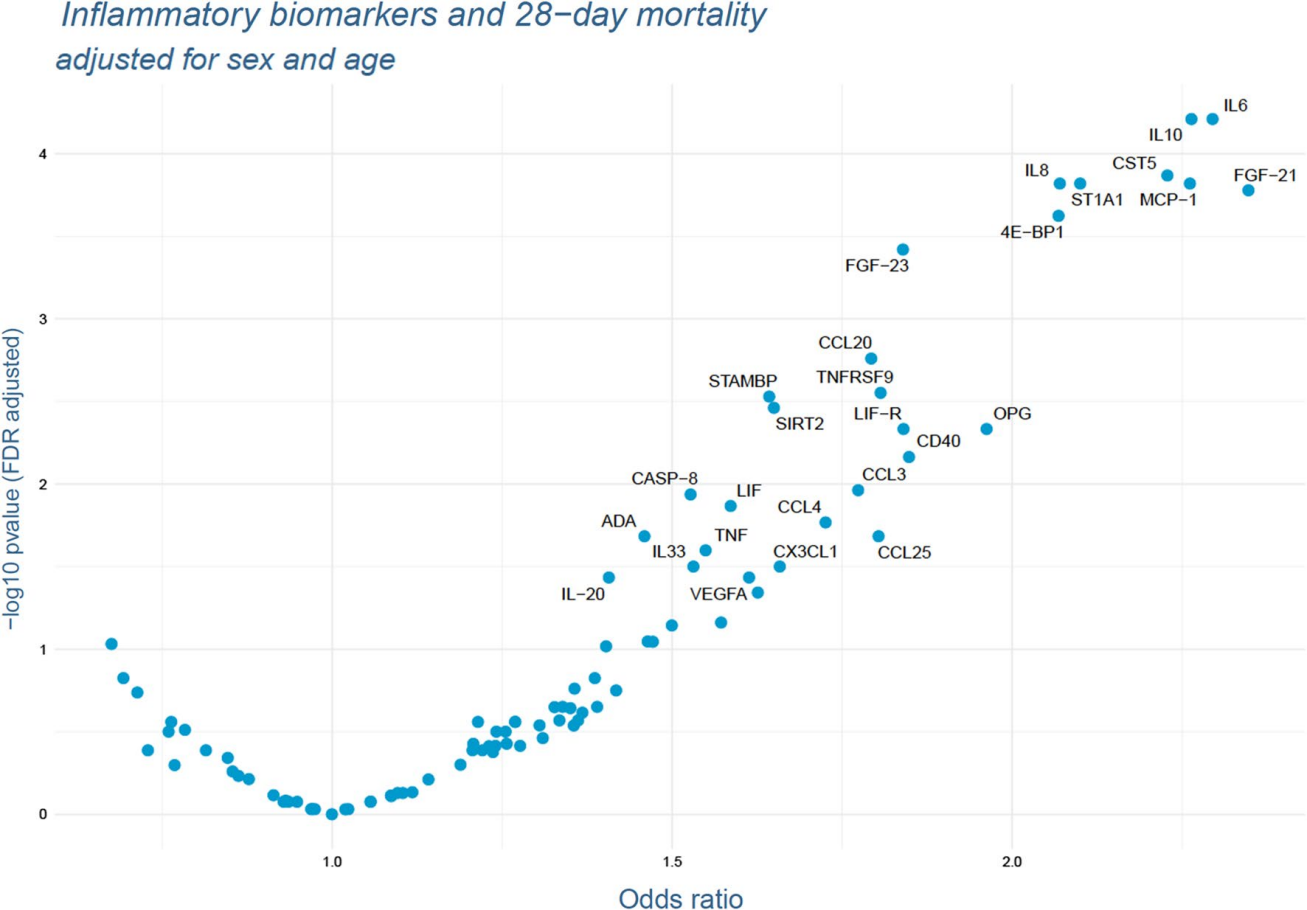
Results of the logistic regression models adjusted for sex and age. P-values were FDR-adjusted. Names of the markers are presented for all markers with FDR-adjusted p-values below 0.05

**Fig. 2:**
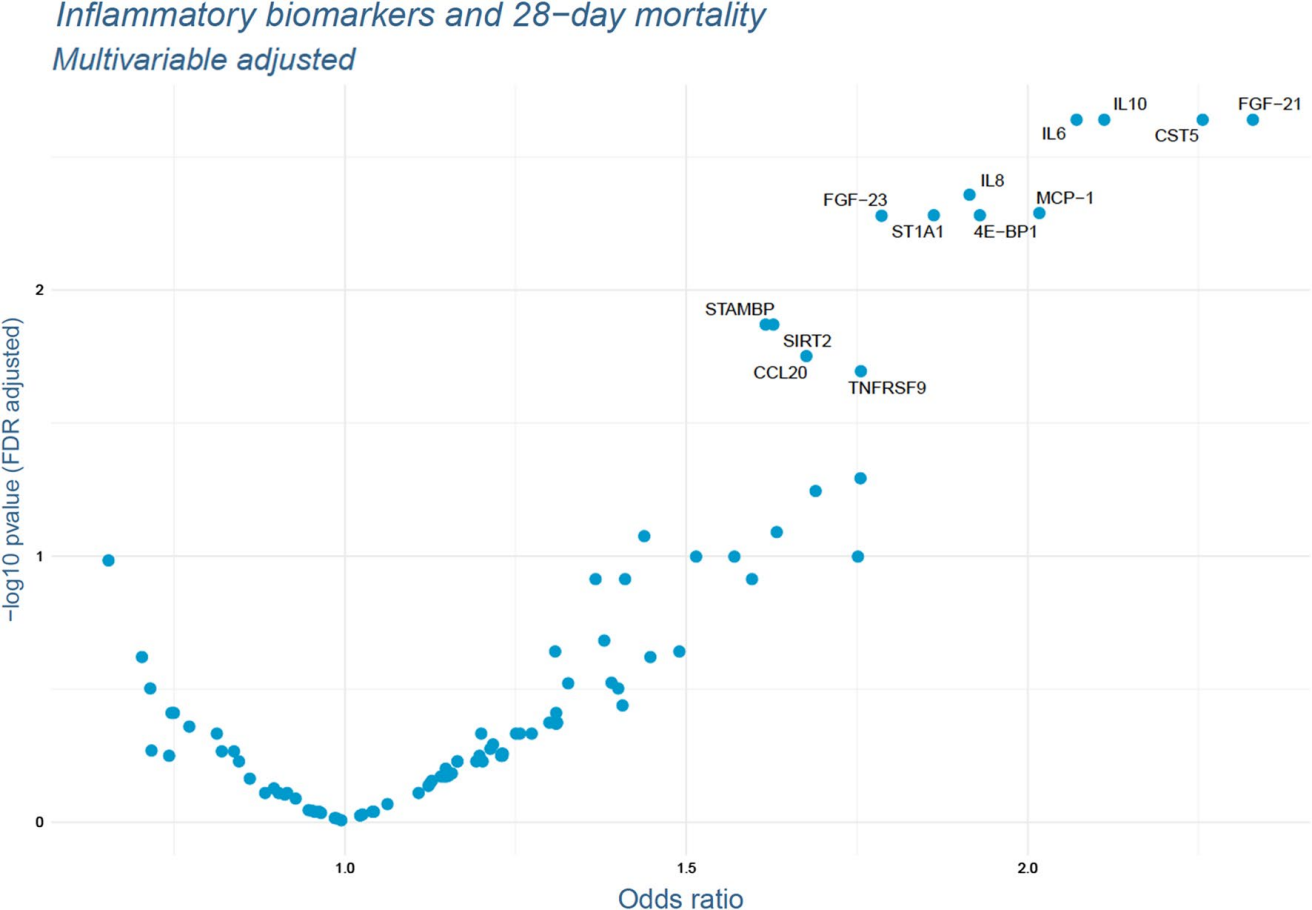
Results of the multivariable adjusted logistic regression models. P-values were FDR-adjusted. Names of the markers are presented for all markers with FDR-adjusted p-values below 0.05. Adjusted for: sex, age, renal function according to GFR, diabetes mellitus, hypertension, hyperlipidemia, PCI

Results of the logistic regression model adjusted for sex and age are displayed in Fig. 1. There were 9 protein biomarkers with adjusted p-values < 0.001: IL6 (Interleukin 6), IL10 (Interleukin 10), IL8 (Interleukin 8), CST5 (Cystatin D), MCP-1 (chemoattractant protein-1), ST1A1 (Sulfotransferase 1A1), FGF-21 (Fibroblast growth factor 21), FGF-23 (Fibroblast growth factor 23), and 4E-BP1 (Eukaryotic translation initiation factor 4E-binding protein 1). The results of the multivariable adjusted logistic regression models are displayed in Fig. 2. The same 9 parameters remain producing the strongest p-values < 0.01. For all other parameters, the adjusted p-values were > 0.01. In Table 1 of the supplementary material, the results of the logistic regression models are displayed numerically including the full names of all biomarkers that were measured.

**Fig. 3:**
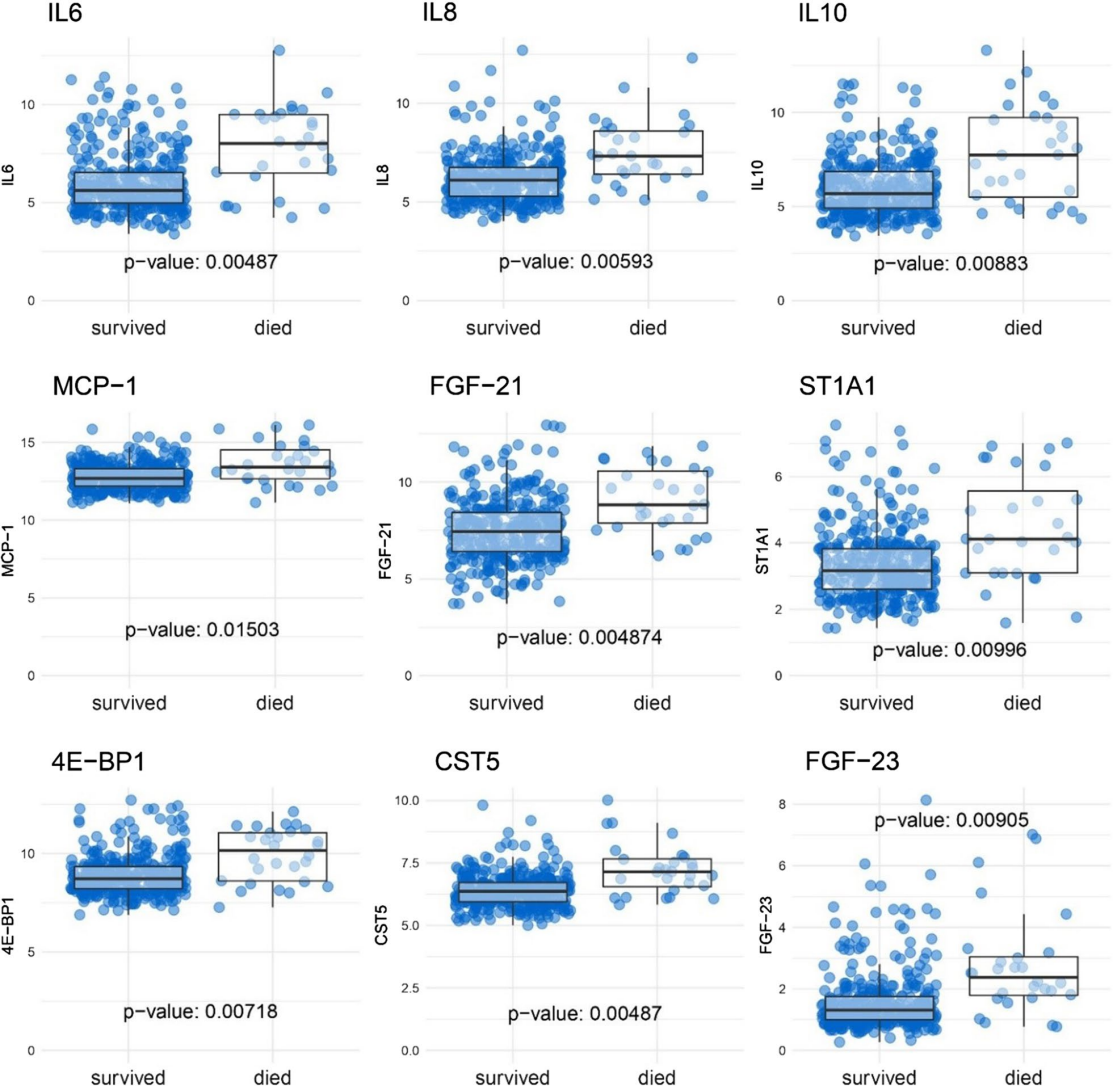
Boxplots of the 9 biomarkers which had the strongest association with 28-day mortality. The plots are stratified for patients who survived the first 28 days and those who did not. For all markers, the values were higher in the group of patients who died within 28 days after AMI. P-values were calculated using t-tests and were FDR-adjusted

The values of 9 parameters identified in the logistic regression models are displayed in Fig. 3. The plots are stratified for 28-day survival. Patients who died within the first 28-days after infarction had higher values for all 9 parameters compared to patients who survived the first 28 days.

**Fig. 4:**
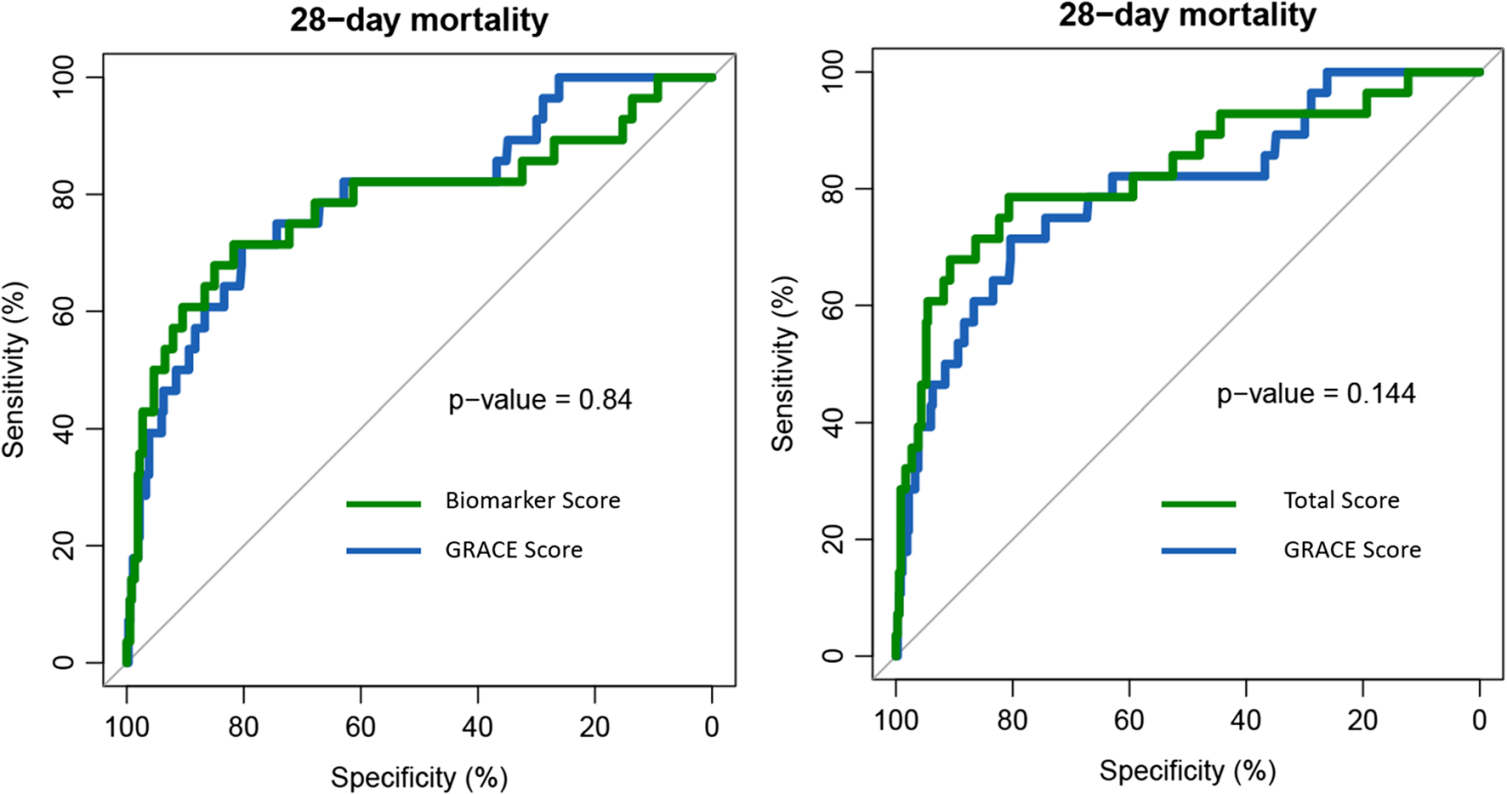
ROC curves for the biomarker score (on the left) and the combined total score (on the right) in comparison to the GRACE score (blue curve). The displayed p-values were obtained from comparing the AUC values via bootstrapping

Figure 4 shows the ROC curves of the biomarker score and the total score in comparison to the GRACE score. For each ROC curve, the number of events (= deaths within 28 days) was 28 and the number of controls was 367. The AUC of the biomarker score was 0.7859 [CI: 0.6735–0.89] and for the GRACE Score 0.7961 [CI: 0.6965–0.8802] (p-value for difference in AUC values: 0.84). The AUC of the combined total score was 0.8305 [CI: 0.7269–0.9187] (p-value for difference in AUC values compared to GRACE score: 0.144). The comparison of the GRACE score with the biomarker score revealed no significant superiority of one score over the other with a cNRI of 0.113 (CI: -0.268–0.493, p-value: 0.561) and an IDI of 0.047 ((CI: -0.033–0.127, p-value: 0.25). The combined total score on the other hand showed a significantly better discrimination compared to the GRACE score alone with a cNRI of 0.566 (CI: 0.192–0.94, p-value: 0.003) and an IDI of 0.083 (CI: 0.016–0.149, p-value: 0.015). Table 2 displays the AUC values for the individual biomarkers (ranging from 0.6914 [MCP-1] up to 0.7713 [FGF-23]); confirming the results of the logistic regression models by proofing a very good discrimination for each individual biomarker. In the supplementary material we report the AUC values for the individual components of the GRACE score (Additional file 1: Table S2).

**Table 2:** Results of the ROC analyses for each individual biomarker

Biomarker	AUC [95% CI]
IL-6	0.7431 [0.6181–0.8516]
IL-8	0.7513 [0.6535–0.8434]
IL-10	0.7087 [0.5913–0.8215]
FGF-21	0.7461 [0.6518–0.836]
FGF-23	0.7713 [0.6699–0.866]
ST1A1	0.7169 [0.5992–0.8243]
MCP-1	0.6914 [0.5747–0.807]
4E-BP1	0.7183 [0.5973–0.8284]
CST5	0.767 [0.6648–0.8653]

Since for every patient included in this analysis a cardiac catheterization was performed, but not every patient was treated by PCI, a subgroup analysis including only patients who received PCI treatment was performed (supplementary material: logistic regression models: Additional file 1: Figure S2; ROC analyses: Additional file 1: Figure S3). Even though the results indicated very slightly attenuated associations between the biomarkers and 28-day mortality, the general association remains significant confirming the results obtained by the analyses using the total sample.

## Discussion

In this study, we investigated the association of 92 biomarkers with short-term mortality, i.e. death of any cause in the first 28-days, in acute ST-elevation myocardial infarction patients. The biomarkers most significantly associated with short-term mortality were IL-6, IL-10, IL-8, MCP-1 (mainly inflammatory markers), FGF-21, ST1A1, 4E-BP1 and CST5 (classified by OLINK as cardiovascular markers) and FGF-23 (classified as a cancer marker). For some of these nine markers prior studies also found associations with short-term mortality in AMI patients, in particular IL-6, FGF-23 and FGF-21 (see below).

### Interleukins IL-6, IL-8 and IL-10

While the interleukins IL-6 and IL-8 are classified as pro-inflammatory cytokines [15, 16]; IL-10 is suspected to have predominantly anti-inflammatory effects [17]. A systematic review by Kristono et al. summarized a potential association between inflammatory cytokines and long-term adverse outcomes in acute coronary syndromes, including studies analyzing the cytokines IL-6, IL-8 and IL-10 [18]. They concluded that some studies reported significant associations between individual cytokines (including IL-6, IL8 and IL-10) and MACE (major adverse cardiovascular events); but they also found considerable heterogeneity in the methods and results of the studies included their review [18]. They furthermore suggested that a combination of multiple cytokines might have a better association with MACE than individual cytokines [18].

Many potential pathophysiological pathways, which might convey a potential association between these cytokines and the outcome of AMI patients are described in the scientific literature. ***IL-6*** for example is more and more commonly used as a long-term marker of inflammation. It is suspected to be a cardiovascular risk factor and to be correlated with endothelial dysfunction and subclinical atherosclerosis [19]. As an example, the authors of a meta-analysis concluded, that chronic use of phosphodiesterase inhibitors in in type 2 diabetes mellitus patients has a beneficial effect on endothelial function conceivably by a reduction of IL-6 serum levels [20]. For ***IL-8***, a study by Shetelig et al. found that high levels were associated with larger infarct size and lower LV ejection fraction in STEMI patients [21]. In a study on ***IL-10***, Jung et al. claimed, that IL-10 improves cardiac remodeling after AMI by stimulating M2 macrophage polarization and fibroblast activation [22]. The authors of another study concluded, that IL-10 inhibits inflammation and attenuates left ventricular remodeling after myocardial infarction via activation of STAT3 and suppression of HuR [23]. Further studies suggested a multitude of other biochemical pathways that might be involved in the complex relationship between these three cytokines and myocardial infarction [24–32].

### Fibroblast growth factor family

In the present study, two representatives of the fibroblast growth factor family, FDF-21 and FDF-23, were highly associated with 28-day mortality after AMI. Prior publications on ***FGF-23*** suggested an association between elevated FGF-23 levels and cardiovascular events in general and mortality in CAD in particular [33–37]. Though, the authors of a meta-analysis on this topic concluded, that the association might be non-causal [38]. In a rat model, Andrukhova et al. found that the induction of myocardial infarction in rats led to an elevation of FGF-23 levels [39], which strongly indicates a general association between AMI and FGF-23. Though, results of their study might rather suggest that AMI causes an elevation of FGF-23 levels and not the other way round. The results of the presents study would further indicate, that FGF-23 elevations are not only associated with the event itself, but also with adverse outcome after AMI.

For ***FGF-21***, several prior studies suggested, that FGF-21 inhibits inflammation and fibrosis after AMI and therefore might improve cardiac remodeling [40–42]. A review proposed FGF-21 as a new cardiomyokine which is crucial for maintaining cardiac function and has positive effects on the heart in the context of pathological conditions [43]. Therefore, we should have expected to find an inverse correlation between FGF-21 and mortality, which we didn´t. One possible explanation might be that very high levels of FGF-21 indicate great myocardial damage with a pronounced counter-regulation. Therefore despite having a protective effect, high FGF-21 levels are associated with higher mortality since they represent greater myocardial damage. This hypothesis is supported by a small study from China, which revealed significantly higher FGF-21 levels in AMI patients compared to non-AMI control patients, indicating that high FGF-21 levels are independently associated with AMI [44].

### MCP-1, CST5, ST1A1 and 4E-BP1

Another four biomarkers, i.e., MCP-1, CST5, ST1A1 and 4E-BP1, were strongly associated with 28-day mortality and consequently were included in the biomarker score. ***MCP-1*** is a chemokine that regulates migration and infiltration of monocytes/macrophages [45]. A review article on cardiac repair after myocardial infarction reported, that early recruitment of pro-inflammatory monocytes is mediated through activation of the MCP-1/CCR2 axis and that in mice, MCP-1 inhibition exhibits a reduced infarct size and monocyte infiltration [46]. Next to a reduction of IL-6 serum levels as described above, it has been reported that phosphodiesterase-5 inhibitors like sildenafil also reduce MCP-1 levels in men with diabetes [47] and affect circulating monocytes and tissue inflammatory cell infiltration [48]. Another study including 87 AMI patients and 82 controls found higher levels of MCP-1 in the AMI group compared to a control group [49]. Additionally, they reported, that the highest MCP-1 levels were observed in patients with poor prognosis, which agrees with results of the present study.

For the remaining biomarkers ***CST5*** (Cystatin D), ***ST1A1*** and ***4E-BP1*** we couldn´t find prior studies that investigated a potential association between these biomarkers and AMI or AMI-related mortality, so we cannot compare our results to previously reported results. Further studies are needed to confirm the relationship of these newly detected protein biomarkers with adverse outcome after STEMI.

It could be conjectured, that the association between the nine biomarkers and short-term mortality after STEMI might be driven by the delay between symptom onset and PCI: patients with longer therapeutic delay have longer ischemic times which increases the plasma levels of the biomarkers at cardiac catheterization and at the same time adversely affects the outcome after AMI. Therefore we calculated the multivariable adjusted logistic regression models as described in the results section but added prehospital delay as an additional covariable (see Fig. 1, supplementary material). Although this influences the results of the model to a limited extent, it shows that therapeutic delay does not play a superior role in the association between the investigated biomarkers and short-term mortality.

Another important issue to consider is the well-known interconnection of pathological inflammatory processes with obesity and obesity-related diseases such as diabetes mellitus type 2. A general example for this association is an obesity-related increased susceptibility to infectious diseases [50], which also became visible in the context of the Covid-19 pandemic. But there is also increasing evidence that the link between obesity and inflammation plays a major role in metabolic and cardiovascular diseases [51–53]. For instance, in a review paper Tarsitano et al. discussed the specific relation between epicardial adipose tissue and its pro-inflammatory role in several cardiovascular diseases [54]. It has moreover been reported, that specific cardio-protective agents like Phosphodiesterase-5 inhibitors can positively affect the course of the diseases by modifying underlying inflammatory processes [55, 56]. Against this background, one could have assumed that obesity in terms of an elevated BMI might be strongly involved the relationship between the inflammatory biomarkers and short-term mortality after AMI. Nevertheless, in the present study we found almost no differences for BMI between the groups and consequently no evidence indicating and substantial importance of BMI/obesity for the reported associations.

### Discrimination and prediction scores

We found that the predictive ability of our calculated biomarker score is comparable to that of the well-established GRACE score. With AUC values of 0.7859 and 0.7961 both scores showed proper and comparable discrimination between cases (patients who died within 28 days) and controls. When both scores were combined to a total score, the predictive ability could be further increased (AUC of 0.8305) and according to cNRI and IDI the combined score added predictive ability to the GRACE score. Considering the results of the multivariable adjusted logistic regression models, it can be concluded that these nine markers are independently associated with short-term outcome after AMI.

These results may have two major implications. First, these markers could be measured in order to estimate a patient’s risk of adverse outcome, which then could be considered by physicians in therapy decisions. Currently, however, these markers are very rarely determined in the medical context and their measurement is time consuming and expensive, which makes them unfavorable candidates for early risk stratification unless a method for easy determination (e.g., an ELISA test) will be established.

Second, the markers found to have good predictive ability for short-term outcome might not only be associated with 28-day mortality but actually be causally related to mortality after AMI. If this would be the case, tailored treatment and pharmacological interventions might improve the outcome in AMI patients. Foremost, drugs that block the biological marker itself or that act on the corresponding receptors should be considered as a promising approach. Hartman et al. for instance gave a translational overview of anticytokine therapy in cardiovascular disease and after AMI [57]. They concluded, that promising results were mainly seen in experimental studies and in smaller clinical studies, but only one larger RCT showed positive results on outcome so far. But from our point of view the other biomarkers identified in this study would also deserve to be further investigated with regards to potential benefits of medical therapies. Nevertheless, to this day there is a lack of studies and evidence whether such new pharmacological approaches would indeed favorably affect the underlying pathophysiology of CAD and whether new drugs would improve outcome after AMI. The results reported in this study provide new starting points towards drug development and for future personalized treatment of AMI patients. Researchers in this field should strongly feel encouraged to further explore the possibilities that are revealed by this study.

### Strengths and limitation

This study is characterized by some strengths. First, this study is based on patients from the population-based myocardial infarction registry Augsburg with consecutive enrollment, which minimizes the effect of selection bias. Blood samples were uniformly taken immediately before the PCI intervention, guaranteeing highest consistency in blood sampling. Moreover, for every case there was a large number of additional information which we used for proper adjustment in the logistic regression models and allowed us to imitate the established GRACE score to compare predictive abilities.

Nevertheless, there are also some limitations. No validation cohorts from other registers are available for our analyses, preventing validation of the associations—in particular for the newly identified protein markers—found in this study. Moreover, as this study is based solely on observational data, we cannot draw any conclusions about causality (including the possibility of reverse causality). Even though the 92 markers measured in this study cover a broad spectrum of important and promising inflammatory markers, there are more inflammatory markers and plasma proteins that may have been of great importance in this context. Additionally, we might not have considered all relevant confounders. Furthermore, as this study included only STEMI patients with age between 25 and 84 years, the results may not be generalized to all age groups or ethnic groups as well as to Non-ST-elevation ACS events. Finally, as majority of the study cohort was male, it is not entirely clear whether the results can also be applied to female AMI patients. Prior studies have shown important differences in pathophysiology and response to treatment for both sexes in cardiovascular diseases [58, 59]; though the number of female patients in this study is insufficient for a valid subgroup analysis to address this limitation.

## Conclusion

Several inflammatory protein biomarkers were strongly related to adverse outcome after AMI. Combined to a biomarker score, they showed proper predictive ability for 28-mortality after ST-elevation myocardial infarction. Adding the biomarker to the established GRACE score improved the discrimination with an AUC of 0.8305. Even though conclusions about a causal relationship cannot be drawn, these biomarkers might provide novel possibilities for drug development and individualization of therapy regimes in STEMI patients.

## Supplementary Information

**Additional file 1: Table S1.** Results of the logistic regression models including the biomarkers full names. **Table S2.** Predictive ability of the individual components of the GRACE score. For each component, a ROC analyses for 28-day mortality was calculated and its results are displayed in the table below. **Figure S1**. Multivariable adjusted logistic regression models including the additional covariate ‘prehospital time’. P-values were FDR-adjusted. Names of the markers are presented for all markers with FDR-adjusted p-values below 0.05. **Figure S2**. Multivariable adjusted logistic regression models including only observations of patients who received PCI treatment. P-values were FDR-adjusted. Names of the markers are presented for all markers with FDR-adjusted p-values below 0.05. **Figure S3**. ROC curves for the biomarker score (on the left) and the combined total score (on the right) in comparison to the GRACE score (blue curve). Only cases of patients who received PCI treatment were included. The displayed p-values were obtained from comparing the AUC values via bootstrapping.

## Data Availability

The datasets generated during and/or analyzed in the current study are not publicly available due to data protection aspects but are available in an anonymized form from the corresponding author on reasonable request.

## Abbreviations

AMI: Acute myocardial infarction
4E-BP1: Eukaryotic translation initiation factor 4E-binding protein 1
AUC: Area under the curve
CAD: Coronary artery disease
cNRI: Continuous net reclassification improvement
CST5: Cystatin D
FDR: False discovery rate
FGF: Fibroblast growth factor
IDI: Integrated discrimination increment
IL: Interleukin
IQR: Inter-quartiles range
KORA: Kooperative Gesundheitsförderung in der Region Augsburg
LVEF: Left ventricular ejection fraction
MACE: Major adverse cardiovascular events
MCP-1: Chemoattractant protein-1
MONICA: Monitoring Trends and Determinants in Cardiovascular disease
NT-proBNP: N-terminal pro B-type natriuretic peptide
PCI: Percutaneous coronary intervention
SD: Standard deviation
ST1A1: Sulfotransferase 1A1
STEMI: ST-elevation myocardial infarction
