# Supplementary material for: Inflammatory plasma proteins predict short-term mortality in patients with an acute myocardial infarction
Source: J Transl Med. 2022 Oct 8;20:457. doi: 10.1186/s12967-022-03644-9 (PMC9547640; doi:10.1186/s12967-022-03644-9)
Supplement: Supplementary file 1 — Additional file 1: Table S1. Results of the logistic regression models including the biomarkers full names. Table S2. Predictive ability of the individual components of the GRACE score. For each component, a ROC analyses for 28-day mortality was calculated and its results are displayed in the table below. Figure S1. Multivariable adjusted logistic regression models including the additional covariate ‘prehospital time’. P-values were FDR-adjusted. Names of the markers are presented for all markers with FDR-adjusted p-values below 0.05. Figure S2. Multivariable adjusted logistic regression models including only observations of patients who received PCI treatment. P-values were FDR-adjusted. Names of the markers are presented for all markers with FDR-adjusted p-values below 0.05. Figure S3. ROC curves for the biomarker score (on the left) and the combined total score (on the right) in comparison to the GRACE score (blue curve). Only cases of patients who received PCI treatment were included. The displayed p-values were obtained from comparing the AUC values via bootstrapping. [file 12967_2022_3644_MOESM1_ESM.pdf]

Supplementary material

Inflammatory plasma proteins predict short-term mortality in patients with an acute myocardial infarction

Schmitz T, Harmel E, Heier M, Peters A, Linseisen J, Meisinger C

Protein Measurement

Proteins were measured by Olink Proteomics (Uppsala, Sweden). Protein concentrations of plasma samples were determined using the Pro-seekQ3XXMultiplex Inflammation Panel based on the proximity extension assay (PEA). Further information can be found on the website. The measurement method can be described as follows: This assay uses a pair of specific antibodies labeled with oligonucleotides that target each protein. When two matching oligonucleotides are close to each other, a new protein-specific sequence is formed by proximity-dependent DNA polymerization. The amount of each protein-specific sequence is then quantified by real-time quantitative polymerase chain reaction (qPCR). This method can be used to simultaneously quantify 92 proteins in 96 samples at the same time. Protein concentrations are reported as Normalized Protein Expression (NPX) values, a relative unit used by Olink. It is calculated from Ct values (qPCR) and is on a log2 scale. A difference of 1 NPX corresponds to a doubling of protein concentration. Normalization is performed to minimize variation within and between assays. The limit of detection (LOD) is calculated separately for each Olink assay and sample plate.

GFRACE Score calculation

Since we did not possess all information that was necessary to exactly replicate the GRACE score, we used all data that was available in order to replicate the GRACE score as best as possible. In the following, we explain the calculation that was used for the score we used. It was strongly orientated on the calculations methods describe in this document [1].

Variable 1: Age: This variable was replicated exactly using the following assignments:

Age (years)	Number of points
<35	0

35-45	(Age-35)*(1.8)
45-55	18 + (Age-45)*(1.8)
55-65	36 + (Age-55)*(1.8)
65-75	54 + (Age-65)*(1.9)
75-85	73 + (Age-75)*(1.8)
85-90	91 + (Age-85)*(1.8)
≥90	100

Variable 2: Heart rate: This variable was also exactly replicated using the following assignments:

HR (bpm)	Number of points
<50	0
50-60	(HR-50)*(3/10)
60-70	3 + (HR-60)*(3/10)
70-80	6 + (HR-70)*(3/10)
80-90	9 + (HR-80)*(3/10)
90-100	12 + (HR-90)*(3/10)
100-110	15 + (HR-100)*(3/10)
110-150	18 + (HR-110)*(12/40)
150-200	30 + (HR-150)*(16/50)
≥200	46

Variable 3: systolic blood pressure: This variable was replicated exactly as well. The following assignments were used:

sBP (mmHg)	Number of points
<80	58
80-100	58 - (sBP-80)*(10/20)
100-110	48 - (sBP-100)*(5/10)
110-120	43 - (sBP-110)*(4/10)
120-130	39 - (sBP-120)*(5/10)
130-140	34 - (sBP-130)*(5/10)
140-150	29 - (sBP-140)*(5/10)
150-160	24 - (sBP-150)*(5/10)
160-180	19 - (sBP-160)*(9/20)
180-200	10 - (sBP-180)*(10/20)
≥200	0

Variable 4: Creatinine: Once more, this variable was replicated exactly according to the following table:

**Creatinine mg/dL**	**Number of points**
<0.2	(Creatinine-0)*(1/0.2)
0.2-0.4	1 + (Creatinine-0.2)*(2/0.2)
0.4-0.6	3 + (Creatinine-0.4)*(1/0.2)
0.6-0.8	4 + (Creatinine-0.6)*(2/0.2)
0.8-1.0	6 + (Creatinine-0.8)*(1/0.2)
1.0-1.2	7 + (Creatinine-1.0)*(1/0.2)
1.2-1.4	8 + (Creatinine-1.2)*(2/0.2)
1.4-1.6	10 + (Creatinine-1.4)*(1/0.2)
1.6-1.8	11 + (Creatinine-1.6)*(2/0.2)
1.8-2.0	13 + (Creatinine-1.8)*(1/0.2)
2.0-3.0	14 + (Creatinine-2.0)*(7/1)
3.0-4.0	21 + (Creatinine-3.0)*(7/1)
≥4.0	28

Variable 5: Killip class: We did not have the exact information used for this classification. It classifies symptoms of acute heart failure after AMI using 4 categories. We only possessed data on left ventricular ejection fraction (LVEF), which does not assess heart failure from a symptom-oriented perspective but rather a heart function oriented perspective. Nevertheless, both are legitimate methods to assess heart function and heart failure and are supposedly highly correlated. We used the following table for assignment:

Killip Class	LVEF	Number of points
I class	Normal (>50%)	0
II class	Mildly reduced (41-50%)	20
III class	Moderately reduced (31-40%)	39
IV class	Strongly reduced (≤ 30%)	59

Variable 6: ST deviation: As our sample consisted of only AMI events that were classified as ST-elevation myocardial infarctions, this variable was set 1 for each case.

Variable 7: Elevated cardiac enzymes: We used measured levels of Troponin I to calculate this variable. Troponin T was barely measured and CK-MB levels were very incomplete. Troponin I on the other hand was available for the vast majority of cases. We used the cut off values of the laboratory that performed the measurement (high-sensitive Troponin I, reference: 0-14 pg/ml)

Troponin I value	Assignment
0-14 pg/ml	0
> 14 pg/ml	1

Variable 8: Cardiac arrest at presentation: This information was not exactly recorded by the registry. Nevertheless, there is a variable with information on prehospital cardiac arrest (yes/no). As both definitions are not exactly equivalent but very closely resemble each other, we used this variable for the score.

Prehospital cardiac arrest	Assignment
no	0
yes	1

The final score was then calculated using the following formula:

*GRACE score = Age + Heart rate + systolic blood pressure + Creatinine + (28 * ST deviation) + LVEF + (14 * elevated cardiac enzymes) + (39 * prehospital cardiac arrest)*

Table S1: *Results of the logistic regression models including the biomarkers full names*

Biomarker		***logistic regression***					
		adjusted for sex and age			***multivariable adjusted***		
**Short name**	**Full name**	**Odds ratio**	**FDR-adjusted p-value**	**-log$_{10}$p-value (FDR adjusted)**	**Odds ratio**	**FDR-adjusted p-value**	**-log$_{10}$p-value (FDR adjusted)**
ADA	Adenosine Deaminase (ADA)	1,459251	2,07E-02	1,683661	1,367166	1,22E-01	0,913469
ARTN	Artemin (ARTN)	1,019437	9,32E-01	0,030549	1,025778	9,35E-01	0,029182
AXIN1	Axin-1 (AXIN1)	1,206477	4,09E-01	0,387762	1,127364	6,99E-01	0,155648
Beta-NGF	Beta-nerve growth factor (Beta-NGF)	1,214458	2,76E-01	0,559639	1,199736	4,64E-01	0,333048
CASP-8	Caspase-8 (CASP-8)	1,527136	1,16E-02	1,936573	1,438416	8,42E-02	1,074817
CCL19	C-C motif chemokine 19 (CCL19)	1,338905	2,23E-01	0,650865	1,256334	4,64E-01	0,333048
CCL20	C-C motif chemokine 20 (CCL20)	1,792795	1,74E-03	2,759372	1,67585	1,77E-02	1,751456
CCL23	C-C motif chemokine 23 (CCL23)	1,368019	2,42E-01	0,615585	1,201766	5,91E-01	0,228623
CCL25	C-C motif chemokine 25 (CCL25)	1,803536	2,07E-02	1,683661	1,596185	1,22E-01	0,913469
CCL28	C-C motif chemokine 28 (CCL28)	0,853616	5,50E-01	0,259853	0,896117	7,46E-01	0,127159
CCL3	C-C motif chemokine 3 (CCL3)	1,773569	1,09E-02	1,962339	1,632462	8,12E-02	1,090194
CCL4	C-C motif chemokine 4 (CCL4)	1,725633	1,71E-02	1,767309	1,570443	1,00E-01	0,9981
CD40	CD40L receptor (CD40)	1,848334	6,86E-03	2,163473	1,751388	1,00E-01	0,9981
CDCP1	CUB domain-containing protein 1 (CDCP1)	1,236527	4,20E-01	0,377	1,108009	7,76E-01	0,109889
CXCL1	C-X-C motif chemokine 1 (CXCL1)	1,49951	7,18E-02	1,143865	1,29955	4,22E-01	0,374333
CXCL10	C-X-C motif chemokine 10 (CXCL10)	1,230773	3,87E-01	0,412713	1,14053	6,73E-01	0,171949
CXCL11	C-X-C motif chemokine 11 (CXCL11)	1,220989	4,09E-01	0,387762	1,145101	6,73E-01	0,171949
CXCL5	C-X-C motif chemokine 5 (CXCL5)	0,814449	4,09E-01	0,387762	0,715062	3,14E-01	0,502609
CXCL6	C-X-C motif chemokine 6 (CXCL6)	1,350406	2,28E-01	0,642838	1,192415	5,91E-01	0,228623
CXCL9	C-X-C motif chemokine 9 (CXCL9)	1,355352	2,90E-01	0,537769	1,40661	3,64E-01	0,438826

CST5	Cystatin D (CST5)	2,228241	1,36E-04	3,867657	2,256201	2,29E-03	2,639662
DNER	Delta and Notch-like epidermal growth factor-related receptor (DNER)	0,713556	1,83E-01	0,737731	0,749729	3,89E-01	0,41057
CCL11	Eotaxin (CCL11)	1,361724	2,70E-01	0,5689	1,230812	5,63E-01	0,249667
4E-BP1	Eukaryotic translation initiation factor 4E-binding protein 1 (4E-BP1)	2,068244	2,38E-04	3,62331	1,929884	5,24E-03	2,280795
FGF-19	Fibroblast growth factor 19 (FGF-19)	1,056356	8,39E-01	0,076467	0,985287	9,65E-01	0,015653
FGF-21	Fibroblast growth factor 21 (FGF-21)	2,347462	1,67E-04	3,777974	2,32973	2,29E-03	2,639662
FGF-23	Fibroblast growth factor 23 (FGF-23)	1,839416	3,81E-04	3,419399	1,785977	5,26E-03	2,278908
FGF-5	Fibroblast growth factor 5 (FGF-5)	1,141635	6,15E-01	0,211407	1,156641	6,55E-01	0,183431
Flt3L	Fms-related tyrosine kinase 3 ligand (Flt3L)	1,571952	6,90E-02	1,161172	1,390507	2,99E-01	0,524226
CX3CL1	Fractalkine (CX3CL1)	1,658313	3,16E-02	1,500252	1,489904	2,28E-01	0,641582
GDNF	Glial cell line-derived neurotrophic factor (GDNF)	0,862239	5,85E-01	0,232905	0,844975	5,91E-01	0,228623
HGF	Hepatocyte growth factor (HGF)	0,877516	6,12E-01	0,213553	0,915559	7,76E-01	0,109889
IFN-gamma	Interferon gamma (IFN-gamma)	0,948447	8,39E-01	0,076022	0,952337	9,06E-01	0,043016
IL-1 alpha	Interleukin-1 alpha (IL-1 alpha)	0,846514	4,55E-01	0,341927	0,812555	4,64E-01	0,333048
IL10	Interleukin-10 (IL10)	2,263612	6,17E-05	4,209401	2,112065	2,29E-03	2,639662
IL-10RA	Interleukin-10 receptor subunit alpha (IL-10RA)	0,931748	8,26E-01	0,082857	0,965092	9,23E-01	0,03479
IL-10RB	Interleukin-10 receptor subunit beta (IL-10RB)	1,023719	9,30E-01	0,031496	0,860805	6,86E-01	0,163851
IL-12B	Interleukin-12 subunit beta (IL-12B)	1,117992	7,34E-01	0,134034	0,955389	9,13E-01	0,039467
IL13	Interleukin-13 (IL-13)	0,729088	4,09E-01	0,387762	0,742661	5,63E-01	0,249667
IL-15RA	Interleukin-15 receptor subunit alpha (IL-15RA)	1,188786	5,01E-01	0,300297	1,040352	9,13E-01	0,039467
IL-17A	Interleukin-17A (IL-17A)	1,269216	2,76E-01	0,559639	1,216924	5,10E-01	0,292328
IL-17C	Interleukin-17C (IL-17C)	1,255062	3,16E-01	0,500753	1,15192	6,68E-01	0,175121
IL18	Interleukin-18 (IL-18)	1,464012	8,98E-02	1,046568	1,309418	3,89E-01	0,41057
IL-18R1	Interleukin-18 receptor 1 (IL-18R1)	1,389716	2,23E-01	0,650865	1,231369	5,53E-01	0,257392
IL2	Interleukin-2 (IL-2)	0,768338	5,03E-01	0,298111	0,716867	5,38E-01	0,269308

IL-2RB	Interleukin-2 receptor subunit beta (IL-2RB)	0,969165	9,30E-01	0,031639	0,994825	9,83E-01	0,007363
IL-20	Interleukin-20 (IL-20)	1,406961	3,68E-02	1,433883	1,307969	2,28E-01	0,641582
IL-20RA	Interleukin-20 receptor subunit alpha (IL-20RA)	0,759512	3,16E-01	0,500753	0,746094	3,89E-01	0,41057
IL-22 RA1	Interleukin-22 receptor subunit alpha-1 (IL-22 RA1)	1,207612	3,74E-01	0,427384	1,147703	6,29E-01	0,201449
IL-24	Interleukin-24 (IL-24)	1,241509	3,16E-01	0,500753	1,164791	5,91E-01	0,228623
IL33	Interleukin-33 (IL-33)	1,531246	3,16E-02	1,500252	1,327011	3,01E-01	0,52198
IL4	Interleukin-4 (IL-4)	0,935829	8,39E-01	0,076467	0,987987	9,67E-01	0,014551
IL5	Interleukin-5 (IL5)	0,928566	8,39E-01	0,076467	0,88322	7,76E-01	0,109889
IL6	Interleukin-6 (IL6)	2,294701	6,17E-05	4,209401	2,071556	2,29E-03	2,639662
IL7	Interleukin-7 (IL-7)	0,99966	9,99E-01	0,000606	0,911993	7,88E-01	0,103733
IL8	Interleukin-8 (IL-8)	2,070095	1,52E-04	3,818846	1,914758	4,39E-03	2,357868
LAP TGF-beta-1	Latency-associated peptide transforming growth factor beta-1 (LAP TGF-beta-1)	1,625984	4,54E-02	1,342763	1,44742	2,39E-01	0,620782
LIF	Leukemia inhibitory factor (LIF)	1,586017	1,36E-02	1,866801	1,410367	1,22E-01	0,913469
LIF-R	Leukemia inhibitory factor receptor (LIF-R)	1,840224	4,65E-03	2,332921	1,689383	5,69E-02	1,244526
CSF-1	Macrophage colony-stimulating factor 1 (CSF-1)	0,970718	9,30E-01	0,031639	0,837662	5,41E-01	0,266422
MMP-1	Matrix metalloproteinase-1 (MMP-1)	1,27647	3,85E-01	0,414744	1,228755	5,63E-01	0,249667
MMP-10	Matrix metalloproteinase-10 (MMP-10)	1,305006	2,89E-01	0,538736	1,229525	5,53E-01	0,257392
MCP-1	Monocyte chemotactic protein 1 (MCP-1)	2,261392	1,52E-04	3,818846	2,017032	5,14E-03	2,28895
MCP-2	Monocyte chemotactic protein 2 (MCP-2)	1,256696	3,74E-01	0,427384	1,124069	7,19E-01	0,143478
MCP-3	Monocyte chemotactic protein 3 (MCP-3)	1,386214	1,50E-01	0,824682	1,197157	5,63E-01	0,249667
MCP-4	Monocyte chemotactic protein 4 (MCP-4)	1,104003	7,43E-01	0,129232	1,04253	9,13E-01	0,039467
CD244	Natural killer cell receptor 2B4 (CD244)	1,086521	7,69E-01	0,11412	0,962333	9,13E-01	0,039467
NT-3	Neurotrophin-3 (NT-3)	1,402828	9,60E-02	1,017557	1,379814	2,08E-01	0,682626
NRTN	Neurturin (NRTN)	1,056685	8,39E-01	0,076467	1,148803	6,73E-01	0,171949
OSM	Oncostatin-M (OSM)	1,334198	2,70E-01	0,5689	1,122343	7,30E-01	0,136583
OPG	Osteoprotegerin (OPG)	1,962426	4,65E-03	2,332921	1,755116	5,10E-02	1,292367
PD-L1	Programmed cell death 1 ligand 1 (PD-L1)	1,471636	9,02E-02	1,044893	1,310915	4,22E-01	0,374333

EN-RAGE	Protein S100-A12 (EN-RAGE)	1,417652	1,78E-01	0,750169	1,309498	4,27E-01	0,369608
SLAMF1	Signaling lymphocytic activation molecule (SLAMF1)	1,35647	1,73E-01	0,761165	1,250618	4,64E-01	0,333048
SIRT2	SIR2-like protein 2 (SIRT2)	1,649695	3,46E-03	2,461013	1,627524	1,35E-02	1,869961
STAMBP	STAM-binding protein (STAMPB)	1,642745	2,96E-03	2,529101	1,616169	1,35E-02	1,869961
SCF	Stem cell factor (SCF)	0,783516	3,08E-01	0,511717	0,702907	2,39E-01	0,620782
ST1A1	Sulfotransferase 1A1 (ST1A1)	2,100051	1,52E-04	3,818846	1,862464	5,24E-03	2,280795
CD6	T cell surface glycoprotein CD6 isoform (CD6)	1,096301	7,43E-01	0,129232	1,062393	8,55E-01	0,068117
CD5	T-cell surface glycoprotein CD5 (CD5)	1,269171	2,75E-01	0,560071	1,16484	5,91E-01	0,228623
CD8A	T-cell surface glycoprotein CD8 alpha chain (CD8A)	0,675613	9,30E-02	1,031571	0,653944	1,04E-01	0,983625
TSLP	Thymic stromal lymphopoietin (TSLP)	0,974498	9,30E-01	0,031496	0,903024	7,76E-01	0,109889
TNFB	TNF-beta (TNFB)	0,913816	7,66E-01	0,115758	0,927964	8,15E-01	0,089008
TRANCE	TNF-related activation-induced cytokine (TRANCE)	0,763408	2,76E-01	0,559639	0,819825	5,41E-01	0,266422
TRAIL	TNF-related apoptosis-inducing ligand (TRAIL)	1,309788	3,46E-01	0,461534	1,273642	4,64E-01	0,333048
TGF-alpha	Transforming growth factor alpha (TGF-alpha)	1,24022	3,85E-01	0,414744	1,022362	9,44E-01	0,024976
TWEAK	Tumor necrosis factor (Ligand) superfamily, member 12 (TWEAK)	0,693014	1,50E-01	0,824682	0,772254	4,37E-01	0,359409
TNF	Tumor necrosis factor (TNF)	1,549256	2,52E-02	1,598092	1,51438	1,00E-01	0,9981
TNFSF14	Tumor necrosis factor ligand superfamily member 14 (TNFSF14)	1,086963	7,76E-01	0,110318	0,947017	9,01E-01	0,04531
TNFRSF9	Tumor necrosis factor receptor superfamily member 9 (TNFRSF9)	1,806539	2,81E-03	2,551726	1,755729	2,02E-02	1,694853
uPA	Urokinase-type plasminogen activator (uPA)	1,327081	2,24E-01	0,649408	1,213091	5,30E-01	0,275721
VEGFA	Vascular endothelial growth factor A (VEGF-A)	1,613109	3,68E-02	1,433883	1,400372	3,14E-01	0,502609

Table S2: *Predictive ability of the individual components of the GRACE score. For each component, a ROC analyses for 28-day mortality was calculated and its results are displayed in the table below.*

Component	***AUC [95% CI]***	***p-value****	***comment***
Age	0.6365 (0.5278-0.7497)	0.0351	AUC is calculated for the continuous variable and not the categorized variable used in the GRACE score
Heart rate at admission	0.5049 (0.3725-0.6336)	<0.001	AUC is calculated for the continuous variable and not the categorized variable used in the GRACE score
Systolic blood pressure at admission	0.7174 (0.6131-0.8131)	0.1689	AUC is calculated for the continuous variable and not the categorized variable used in the GRACE score
Creatinine levels at admission	0.7136 (0.6024-0.8106)	0.1452	AUC is calculated for the continuous variable and not the categorized variable used in the GRACE score
ST deviation	-	-	Not applicable, as the study cohort consisted exclusively of STEMI patients
(Elevated) cardiac enzymes	0.5615 (0.47-0.6385)	<0.001	AUC is calculated for the continuous variable and not the categorized variable used in the GRACE score
Cardiac arrest at presentation	0.6036 (0.5189-0.6941)	0.0013	ROC analysis for categorical variables only makes sense to a limited extent. However, values are displayed for the sake of completeness.
Left ventricular ejection fraction	0.6134 (0.4555-0.7621)	0.0035	ROC analysis for categorical variables only makes sense to a limited extent. However, values are displayed for the sake of completeness.

* in comparison to the biomarker score using bootstrapping

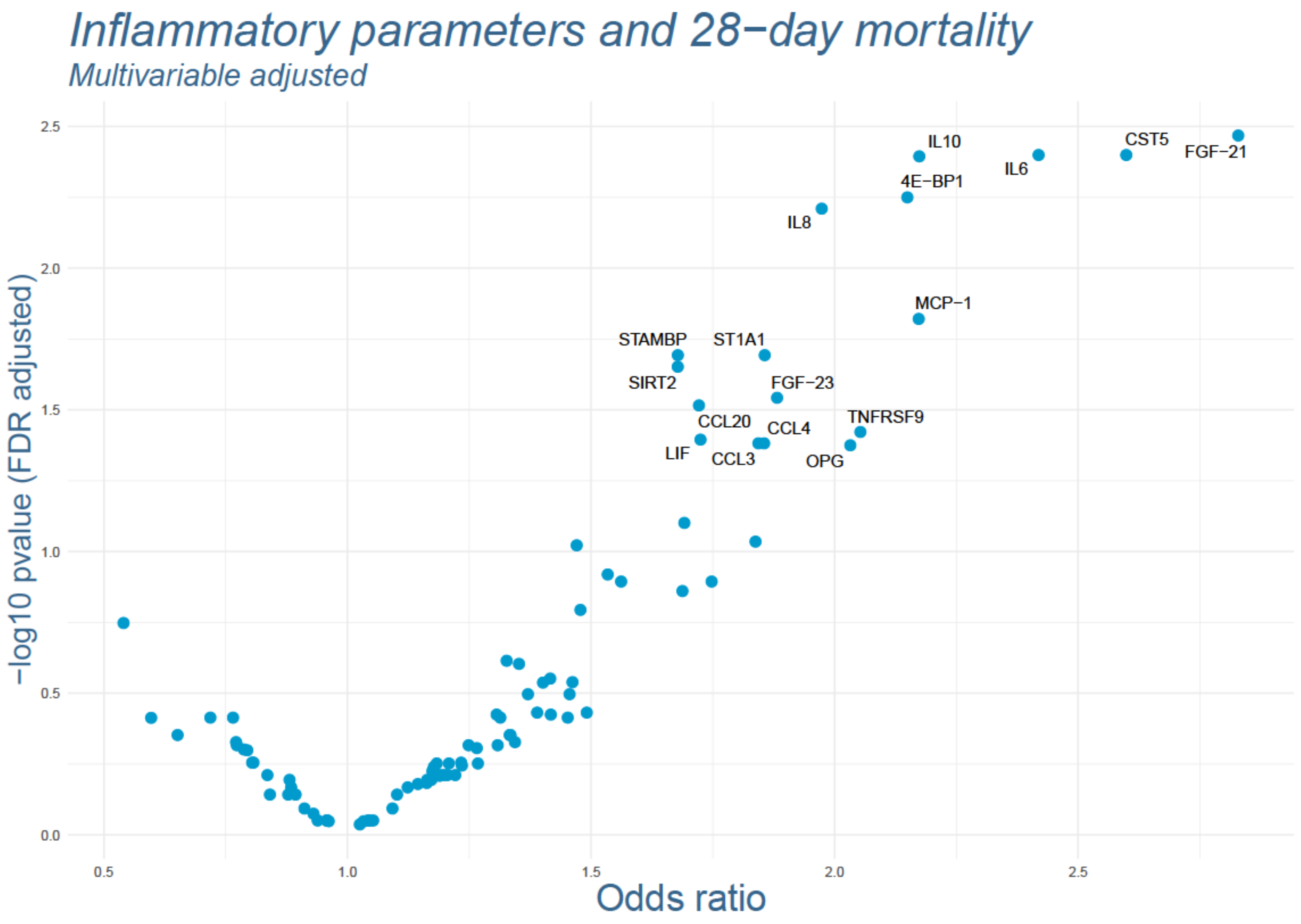

Figure S1: *Multivariable adjusted logistic regression models including the additional covariate 'prehospital time'. P-values were FDR-adjusted. Names of the markers are presented for all markers with FDR-adjusted p-values below 0.05.*

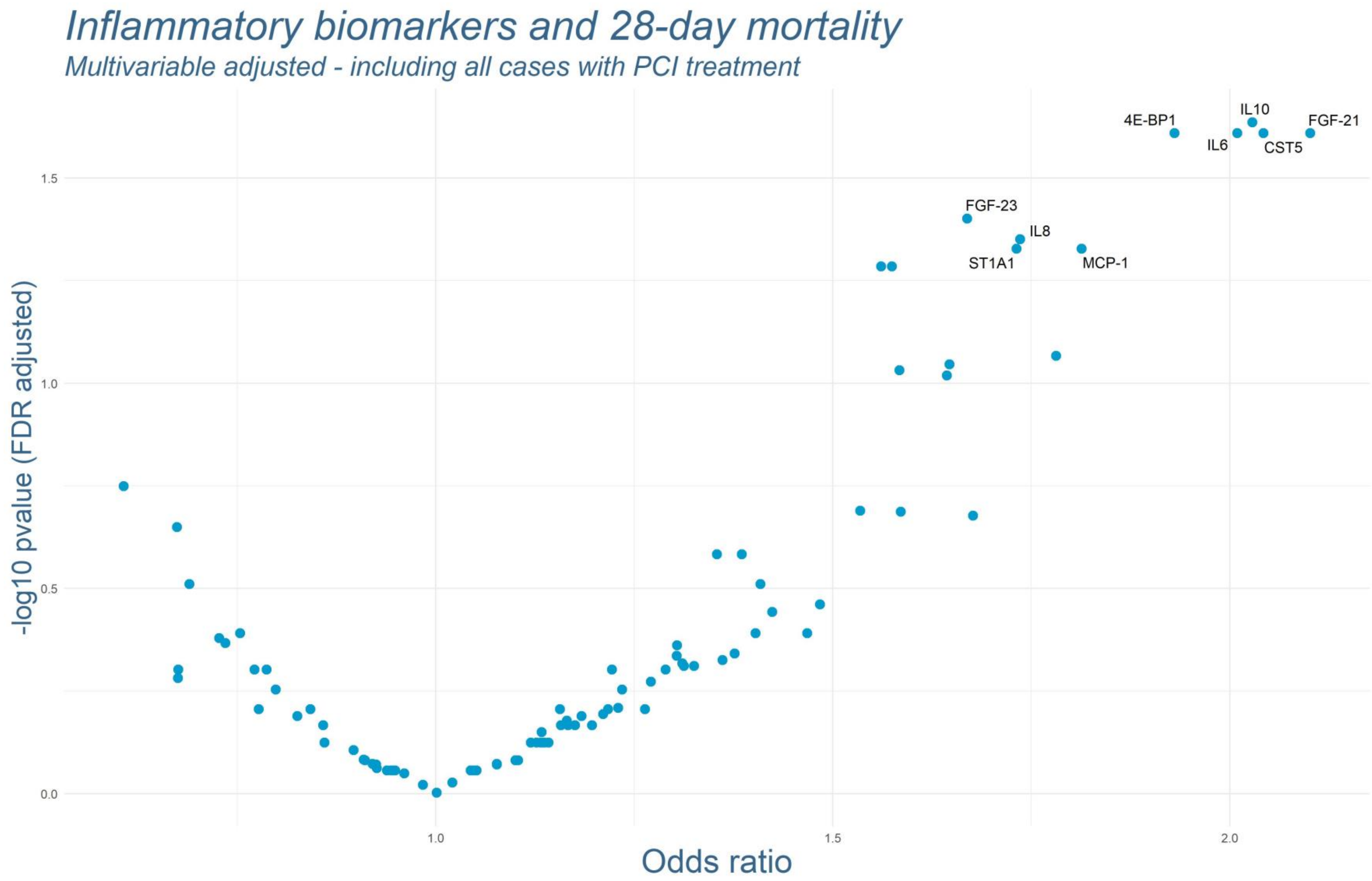

Figure S2: *Multivariable adjusted logistic regression models including only observations of patients who received PCI treatment. P-values were FDR-adjusted. Names of the markers are presented for all markers with FDR-adjusted p-values below 0.05.*

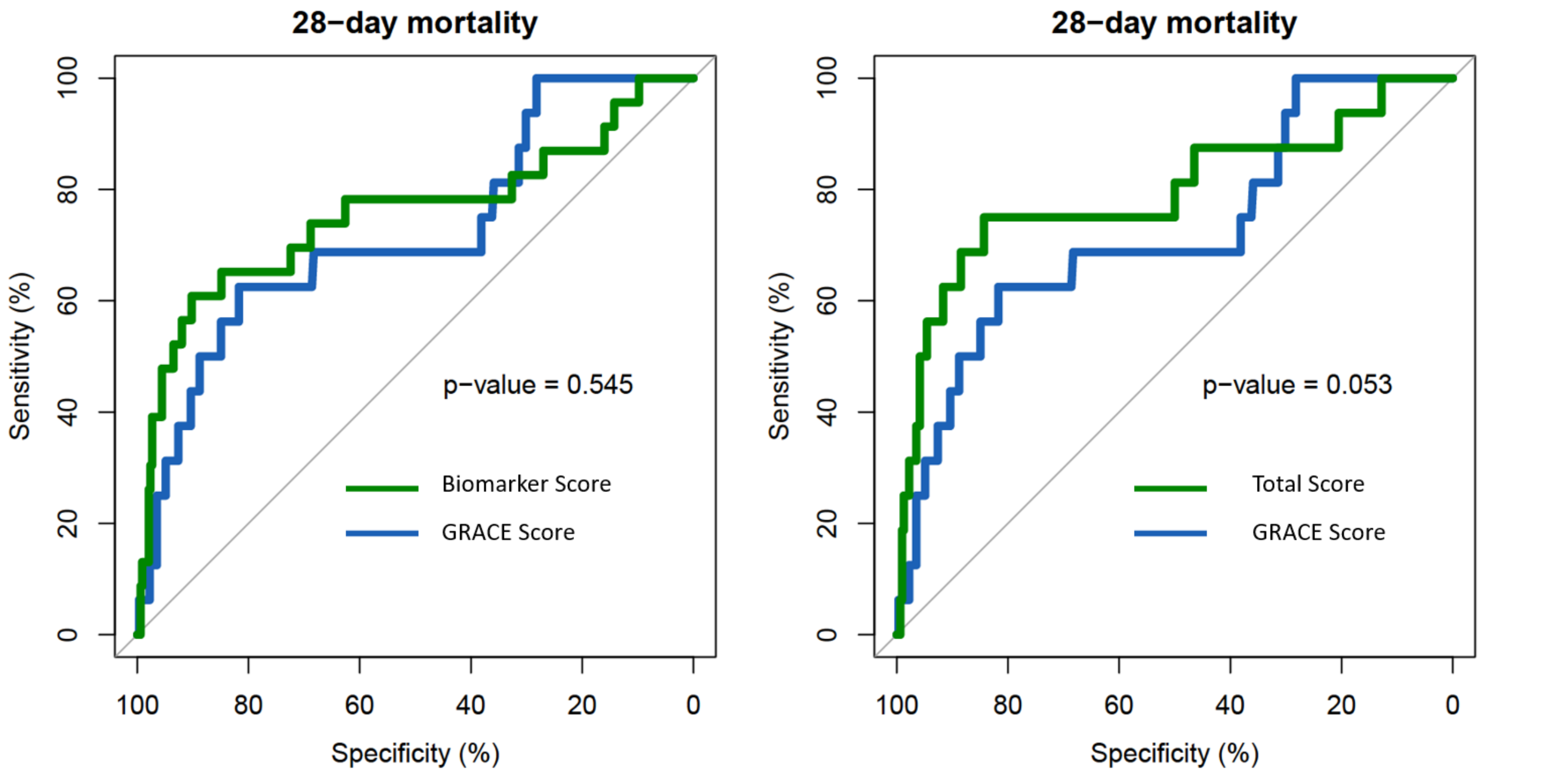

Figure S3: *ROC curves for the* biomarker *score (on the left) and the combined total score (on the right) in comparison to the GRACE score (blue curve). Only cases of patients who received PCI treatment were included. The displayed p-values were obtained from comparing the AUC values via bootstrapping.*

AUC values: Biomarker score: 0.7562 [95% CI: 0.6286-0.8701]; Grace score: 0.7226 [95% CI: 0.5853-0.8522], Total score: 0.7943 [95% CI: 0.6456-0.9231]

REFERENCES

1 Fred Anderson, PhD, Gordon FitzGerald. Methods and formulas used to calculate the GRACE Risk Scores for patients presenting to hospital with an acute coronary syndrome: Updated 2014. Available at: https://www.outcomes-umassmed.org/grace/files/GRACE_RiskModel_Coefficients.pdf.
